# Elaboration of a Movement‐Based Signal in the Presence of a Predator

**DOI:** 10.1002/ece3.73939

**Published:** 2026-07-01

**Authors:** Sonia Khanna, Richard A. Peters

**Affiliations:** ^1^ Animal Behaviour Group, Department of Ecological, Plant & Animal Sciences La Trobe University Melbourne Victoria Australia

**Keywords:** communication, predator, sensory drive, signal modification

## Abstract

Animals balance the need to avoid predators with the requirement to undertake other functional tasks. Animal communication strategies are inherently risky, as they provide information that can be used by eavesdropping predators to detect and localise prey. Given the risks of signalling, most animals modify their signals to reduce detectability during heightened predation risk. We examined signal structure in an Australian lizard ( *Amphibolurus muricatus* ) and predicted predator‐induced changes that would reduce conspicuousness. To do this we facilitated signaller‐intruder exchanges in the immediate presence of a simulated predator and quantified the temporal components of introductory tail flicking and other behavioural measures. Lizards signalled after the intruder was introduced and increased tail flicking time in motion in the presence of the predator, serving to increase signal conspicuousness. Interestingly, lizards also licked the substrate at a greater rate in the presence of the predator. These results contradicted our initial prediction that predator‐induced plasticity primarily favours reduced detectability and suggests that signalling decisions in *A. muricatus* reflect a more complex balance of costs and benefits. We hypothesise that changes were necessary in the present context to overcome the limited attentional capacities of receivers, but the nature of movements ensures the signaller can still visually detect a looming predator and rapidly respond to danger. Although further studies are required, we demonstrate that predator‐induced signal plasticity is multidirectional and may include increases in conspicuousness when necessary to preserve important communication function under threat.

## Graphical abstract

Predation is a driving force in biology and a key determinant of animal behaviour and important functional tasks have been shown to vary in response to changes in the perceived level of threat, including animal communication strategies. Common to all of these is a reduction in the likelihood of detection and/or to minimise exposure to predators. We report on a study that demonstrates the opposite—adjustments made to signal structure by lizards ( *Amphibolurus muricatus* ) when exposed to a predator effectively increase signal conspicuousness.

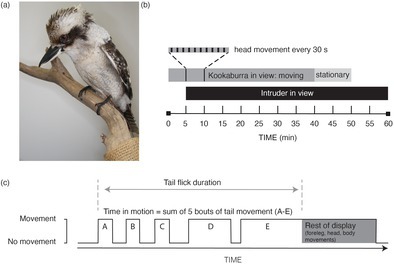

## 1 Introduction

Animals must balance the need to perform functional tasks with staying alive, and an important tool for avoiding predation is to modify behaviour. Predator‐induced adjustments to behaviour are widely reported and include changes to general activity levels (Abbey‐Lee, Mathot, and Dingemanse 2016; Martins et al. 2015), with prey reducing locomotion and increasing refuge use to reduce detection (Lima 1998; Martín and López 2005), or increasing locomotion to flee from an imminent threat (Gemmell et al. 2012; Elmasri et al. 2012; Lohrey et al. 2009). Heightened predation risk also impacts social interactions including reduced aggression (Kelly and Godin 2001), shifts in courtship and mating decisions, including the production of fewer visual displays (Koga et al. 1998; Fowler‐Finn and Hebets 2011), and changes in female preferences (Pilakouta and Alonzo 2014). In addition, prey may also restrict their use of the available microhabitat, such as preferentially using higher perch heights when exposed to ground predators (Losos et al. 2004; Lopez‐Darias et al. 2012; Calsbeek and Cox 2010). Collectively, these responses demonstrate the pervasive influence of predators on behavioural decision‐making and highlight that behaviours increasing conspicuousness, including signalling, are likely to be especially sensitive to predation risk (Abbey‐Lee, Kaiser, et al. 2016; Abbey‐Lee, Mathot, and Dingemanse 2016).

Animal communication strategies are inherently risky as signals should contrast with surrounding conditions to be effective, but predators, who are unintended receivers of intra‐specific signals, can use the information provided to detect and localise potential prey. Predators express strong and consistent preferences for signalling individuals (White et al. 2022) and exploit prey signals through many sensory modalities, including auditory (Tuttle and Ryan 1981), visual (Woods Jr et al. 2007) and electroreception (Stoddard 1999). Multimodal (Halfwerk et al. 2014) and conspicuous signals (Gong 1995; Husak et al. 2006; Stuart‐Fox et al. 2003) are associated with elevated risk as they facilitate the localisation of potential prey. However, there is evidence to suggest that high‐quality individuals can afford to have conspicuous signals, as they are more likely to survive attempted predation events (Hoefler et al. 2008). In contrast, other studies suggest that high‐quality males reduce courtship under risky conditions, suggesting they estimate their long‐term fitness prospects as strong (Rypstra et al. 2016), while lower quality males continue signalling and are willing to suffer higher risks to gain mating opportunities (Moraes et al. 2019). At the population level, high predation pressure can result in natural selection for less conspicuous signals (Giery and Layman 2015), however, individuals can behaviourally modify signalling to decrease risk.

Given these risks, animals have evolved a wide range of behavioural and evolutionary strategies to adjust communication under threat. There are many ways in which animals modify their signalling behaviour, including ceasing or reducing displays, altering signal structure, losing signals and evolving new ones, and increasing signal conspicuousness, although less commonly (Bernal and Page 2023). The cessation of signalling under heightened predation risk has been confirmed in many species (Santema et al. 2019; Lohrey et al. 2009; Akçay et al. 2016; Jones et al. 2002) and underscores the trade‐off animals face between efficient communication and the threat of predation. Populations can also evolve a different signalling modality, such as the loss of conspicuous colour patches and replacement by dynamic motion signals that can be turned off in the presence of unwanted receivers (Martins et al. 2015). In contrast, signallers may benefit from increasing the conspicuousness of sexual signals in risky environments by rapidly securing a mate (Moraes et al. 2019), or producing conspicuous deimatic displays to startle predators and increase chances of escape (Perez‐Martinez et al. 2020; Whiting et al. 2022). Many social prey species have evolved highly conspicuous alarm calls to signal to conspecifics about the presence of predators (Randall et al. 2005) and have even been demonstrated to deceptively mimic the alarm calls of other species to deter predators (Igic et al. 2015). These studies together indicate that the threat of predation has contributed to the evolution of signal form and necessitates plasticity in the communication strategies of animals.

One particularly common class of these adjustments involves modifying signals to reduce detectability or localisability, while maintaining signal efficacy (Marshall and Stevens 2014). Timing signalling to coincide with others is a strategy used by gregarious species to dilute the risk of predation and make it harder to localise individual prey (Brunel‐Pons et al. 2011; Legett et al. 2019). Species can also use private channels that are not perceptible to predators to minimise eavesdropping by unwanted receivers (Cummings et al. 2003; Stoddard 1999). Signals that are intermittent, such as motion displays that can be switched off, minimise detectability by predators (Ord et al. 2021). To further decrease the likelihood of detection, animals can also reduce the amplitude and active space of their signal, and this has been demonstrated in lizards (Simon 2007). Steinberg et al. (2014) found that in response to experimental addition of predators to previously predator‐free islands of the Bahamas, *Anolis sagrei* generated lower amplitude head‐bobs displays relative to lizards on control islands. This serves to reduce the active space of the signal and is the result of an increase in predator abundances over a sustained period. These studies provide increasing evidence of predator‐induced signal plasticity in lizards, and that adjustments are made to parameters of specific components of displays.

We evaluated whether predator‐induced modification to display components is exhibited by the Jacky lizard, *Amphibolurus muricatus* , an Australia dragon lizard with a well‐studied movement‐based signalling system used to display aggressive intent to conspecifics (Peters and Ord 2003). The aggressive territorial displays of *A. muricatus* begin with an introductory tail flicking component, prior to the rest of the display that comprises a sequence of motor patterns, consisting of foreleg waves followed by a push‐up and concluding with a body rock. Of these display components, tail flicking has the largest active space and longest duration and is used to produce an orienting response in intended receivers (Peters and Evans 2003). Importantly, previous research has established that *A. muricatus* are sensitive to the ecological context in which they operate and will extend the duration of introductory tail flicking in the presence of increased plant motion noise (Peters et al. 2007). Signal modification appears to be selective in *A. muricatus* as concomitant changes in tail flicking were not observed in response to different receiver distances (Peters and Allen 2009) as has been shown in crabs *Uca perplexa* (How et al. 2008). There was also no modification to tail flicking by *A. muricatus* when avian predator density was manipulated (Weller 2012), which contrasts with the earlier work on *A. sagrei* (Steinberg et al. 2014).

In this study we consider whether predator‐induced signalling plasticity in *A. muricatus* is evident when there is a more immediate threat by facilitating signaller‐intruder exchanges in the presence of a simulated avian predator perched in proximity. Following the same experimental protocol and analytical strategy as earlier studies to facilitate comparisons (Peters et al. 2007; Peters and Allen 2009; Weller 2012), we quantified the temporal components of introductory tail flicking, alongside other behavioural measures, and predicted that introductory tail flicking displays would be shorter in duration in the presence of a predator, with a reduced time in motion to avoid detection.

## 2 Methods

### 2.1 Study Animals and Animal Housing

**FIGURE A1:**
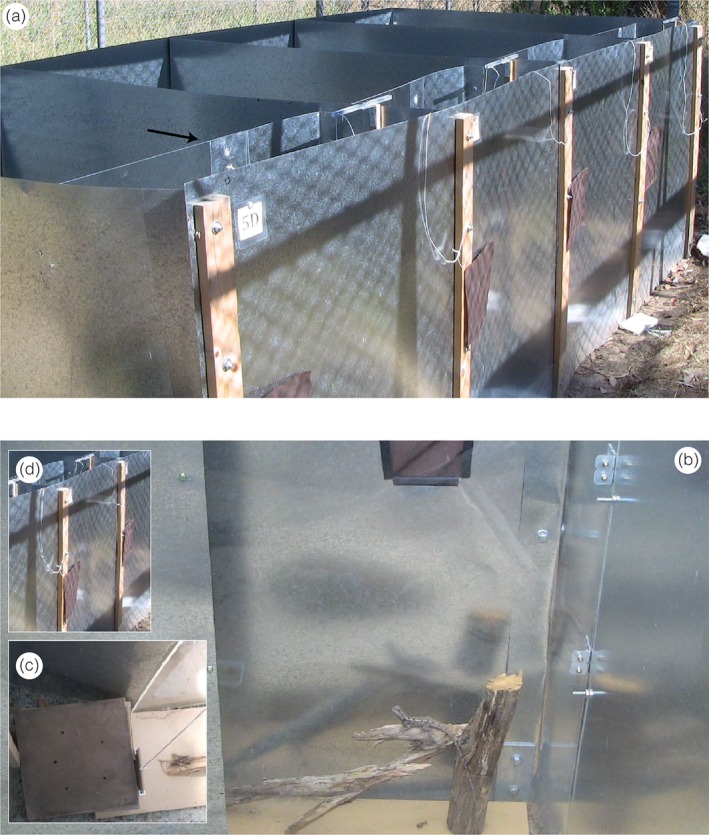
(a) A set of four pens each measuring 1800 × 900 × 1200 mm were constructed from galvanised steel sheets. An internal divider was included in each pen (see arrow) comprised of 300 mm galvanised steel and 600 mm acrylic sheets to physically separate the resident lizard and the intruder lizard (b). The intruder was held in a box until released remotely by use of a string release system (c). The observer sat outside the pen and filmed through a small window covered by cloth (d).

Sixteen adult male *A. muricatus* were caught from Croajingolong National Park, Victoria, Australia and transported to La Trobe University. Two weeks before the experiment these resident lizards were moved into large outdoor enclosures (180 × 90 cm and 120 cm high), constructed from galvanised metal sheets, with a sand substrate and branches for basking. Two sets of four enclosures were used and were designed so that the lizards would be visually isolated from each other, while additional screening ensured lizards could not see what was happening around neighbouring pens. Vegetation was not provided in the testing enclosures. An internal wall of galvanised sheeting (30 cm wide) and clear Perspex (60 cm wide) at one end divided each enclosure into a resident area (floor area of 1.35 m^2^) and an area to present an ‘intruder’ male (0.27 m^2^; Figure A1). Intruder males were caught from the same national park and held indoors in glass reptile enclosures (50 × 50 × 60 cm) with sand substrate, branches for basking and foliage to provide refuge. Heat (Philips Spotone 120 W) and UV lamps (ReptaInfra Plus 60 W) were provided, with lizards fed twice weekly and water available ad libitum.

### 2.2 Predator Model

**FIGURE 1:**
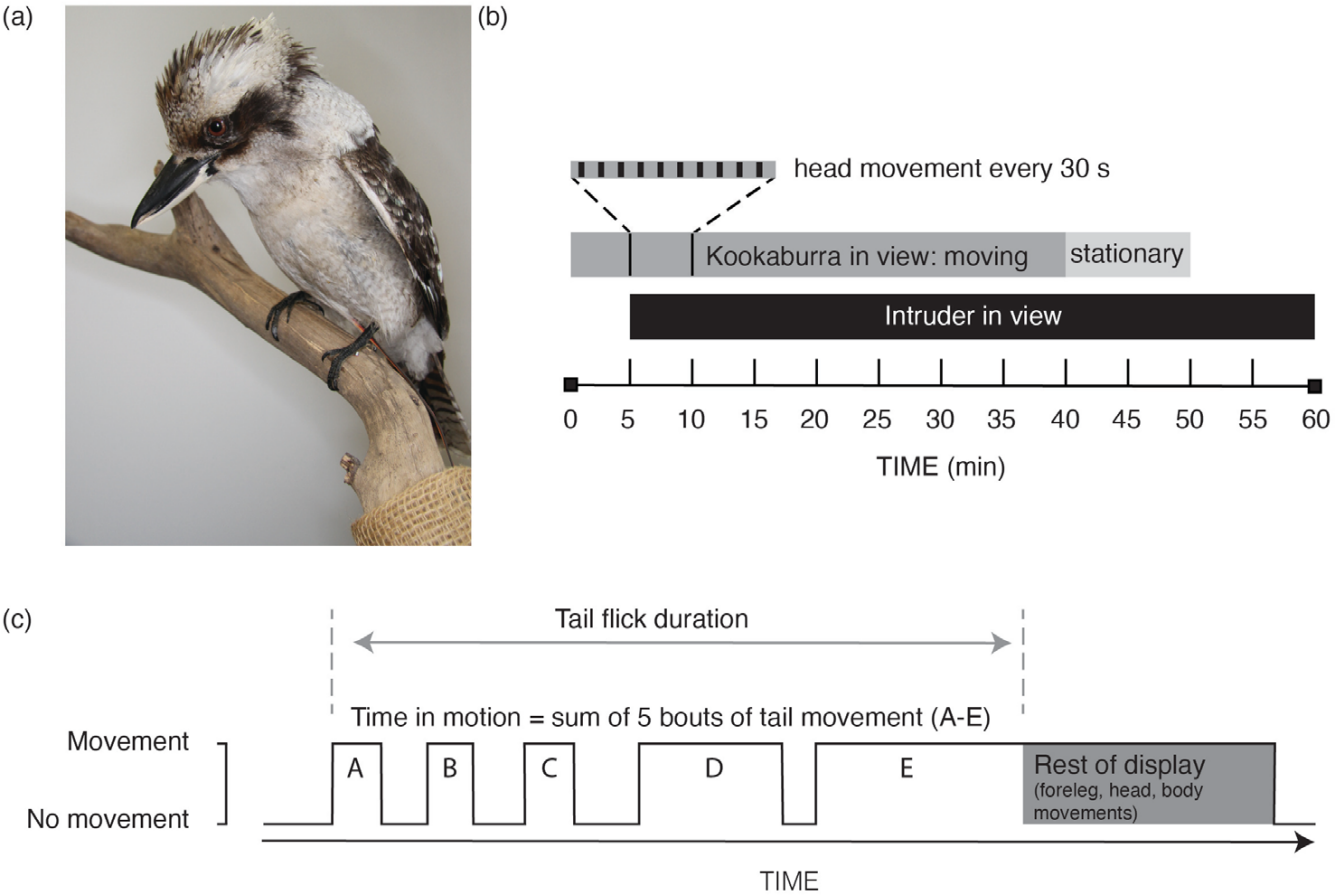
(a) A taxidermic prepared laughing kookaburra fitted with a radio‐controlled servo inside the body enabled movement of the bird's head remotely. (b) The protocol used for presentation of the kookaburra comprised 60 min sessions, with the kookaburra presented for 5 min prior to the release of the intruder. The bird's head was moved left‐and‐right every 30 s for 40 min. (c) Temporal structure of introductory tail flicking by *A. muricatus* comprises intermittent movement. *Duration* was the time between the onset of the first bout of tail movement and the rest of the display; t*ime in motion* was the sum of all bouts of tail movement and r*elative effort* computed from the quotient of *time in motion* over *duration*.

*Amphibolurus muricatus* can be found basking on a variety of substrates with their dorsal patterns matching to varying degrees the patterns of the local substrate (Salisbury and Peters 2025). Avian species that rely on visual detection of prey are the primary threat to adult lizards, with the laughing kookaburra ( *Dacelo novaeguineae* ) being one that co‐occurs across the distribution of *A. muricatus* and is a known predator of the species that uses movement of the lizard to break camouflage (Allen et al. 2009). Thus, for our predator model we used a mounted taxidermy prepared laughing kookaburra perched on a branch (Figure 1a) with modifications to allow small head movements that resemble the natural predatory scanning behaviour of the species. A deceased laughing kookaburra underwent standard preservation techniques to prepare a taxidermy bird specimen. Prior to attaching the head, a small radio control servo (Spektrum A4020, Horizon Hobby Inc.) was inserted into the top of the body and a servo arm fitted inside the bottom part of the head. The servo was connected to a receiver located outside of the bird's body and its movement controlled remotely using a Futaba T2ER transmitter. Accordingly, with the head attached to the body, manual movement of the controller on the transmitter caused the servo in the body to move the servo arm in the head thereby generating turning movements of the head, mimicking the natural scanning behaviour of birds (Fernández‐Juricic 2012). The taxidermy bird was mounted on a branch connected to a wooden frame and secured to a Manfrotto 190XPROB tripod.

### 2.3 Procedure

The experimental design was consistent with earlier studies (Peters and Allen 2009; Peters et al. 2007) to facilitate comparison. Sixteen lizards, in two batches of eight were used in a repeated measures study. Half of each batch of eight lizards received the predator‐present session first followed by the predator‐absent session 4 days later. All sessions were held consecutively in the morning, and the two sessions were at the same time of day for each lizard. A hessian sack was positioned above and to the right of each enclosure and the model moved into place behind the sack. A small box (19.5 × 20 and 8.5 cm) containing a lizard ‘intruder’ was placed within the partitioned off part of the enclosures. A small door at the front of the box connected to a string allowed release of the ‘intruder’ away from view. After 30 min, the hessian sack was removed to reveal the simulated predator. The presentation procedure for the predator‐present sessions from this point is outlined in Figure 1b. Using the radio‐control transmitter, the head of the predator was moved side‐to‐side approximately every 30 s. The door of the intruder container was opened after 5 min to release the intruder. A Canon Legria HF21 camera attached to a tripod filmed the resident lizard through a hole in the wall of the intruder's area, with full coverage of the resident's area. The camera was positioned slightly above the intruder male and, therefore, was at the same height and distance away from the resident male as the intruder. Filming commenced prior to the release of the intruder and continued until the resident male finished its first display bout, or if the resident did not display, within 1 h. Movement of the kookaburra's head was stopped after 40 min if the resident had not performed an aggressive display to see if this change in stimulus condition impacted resident behaviour; however, no signals were performed this late in the session (Figure A3a). Intruder males, selected from a separate captive population, were randomly assigned and size‐matched to within 10% of resident SVL (two pairings were 10%–20%). The procedure for the predator‐absent sessions was the same, with the exception that no predator model was in view when the hessian sack was removed and a different intruder was used. The procedure was subsequently repeated using the second batch of eight lizards.

### 2.4 Video Analysis

Videos were scored using BORIS (Behavioural Observation Research Interactive Software; Friard and Gamba 2016) using a partial ethogram of behaviours relevant to our interests (Table A1). To begin, we wished to determine whether the presence of a simulated predator would affect use of the enclosure and other general behaviours. We scored time spent basking on an elevated perch or on the ground, movement around the enclosure, and when the lizard's eye was oriented upwards. We also examined measures of social behaviour including the use of submissive displays, tail raising, substrate licks, mouth open behaviours and aggressive displays. The introductory tail flicking component of the aggressive display was a central focus. As this component of the display is characterised by intermittent movement (Peters and Evans 2003), from the onset of the first tail movement we recorded bouts of tail movement as well as stationary periods in which the tail did not move (Figure 1c). From this we computed three measures of the temporal structure of tail movements: the time from the first tail movement to the start of the rest of the display (‘tail flick duration’), the time that the tail remained stationary after the initial movement was subtracted from the entire duration to compute the actual ‘time in motion’, while ‘relative effort’ in tail flicking was calculated from the quotient of time in motion and tail flick duration.

### 2.5 Statistical Analysis

**FIGURE A2:**
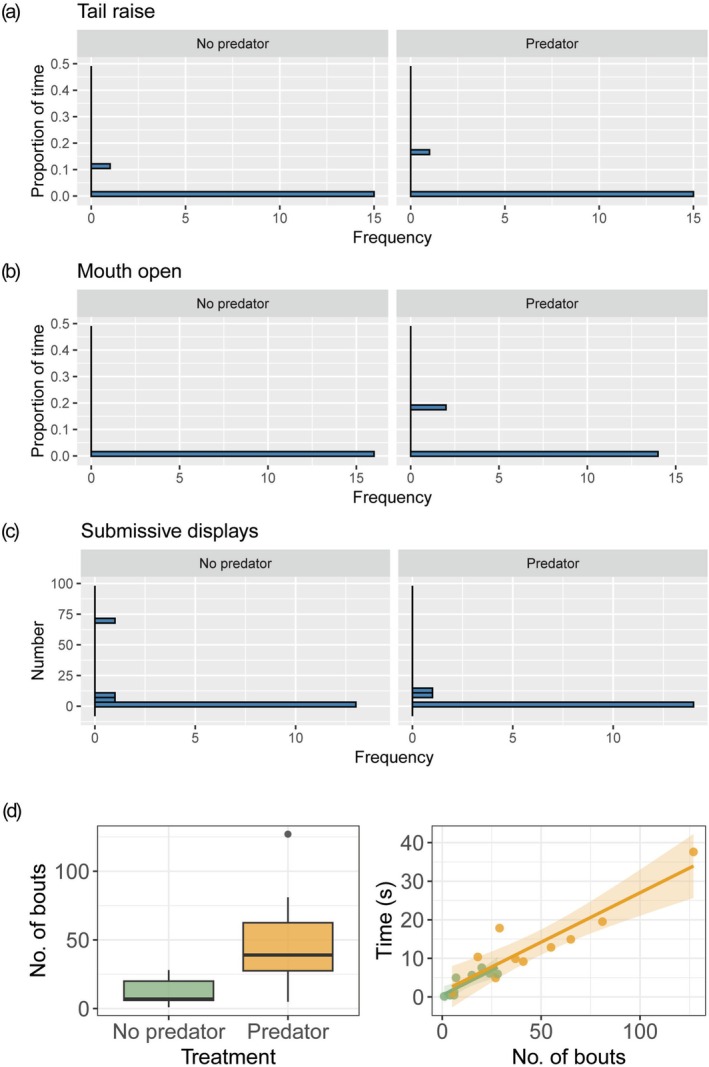
Frequency histograms summarising results for proportion of time lizards exhibiting (a) tail raising and (b) mouth open, as well as the number of submissive displays (c). In (a)‐(c), separate horizontal histograms are shown for no predator (left) and predator (right) conditions. These behaviours were rare and not analysed further. (d) Boxplot showing the number of bouts of tail flicking for the no predator and predator treatments (left panel) and a scatter plot of number of bouts and time in motion for no predator (green) and predator (orange) conditions (right panel).

Statistical analyses were undertaken in the R Statistical Environment (R Core Team 2024). Non‐signalling behaviours of basking, moving and eye up were converted to a proportion of time and analysed using the *glmmTMB* function from the package of the same name (McGillycuddy et al. 2025) as it allows for repeated measures of proportion data. We used treatment (predator present or absent) as a fixed effect and lizard identity as a random effect, setting a beta error distribution with logit link function. Values exactly equal to 0 or 1 were adjusted as per Smithson and Verkuilen (2006) using the formula:

$$p\;\text{transformed}=\frac{p\left(n-1\right)+0.5}{n}$$

where *p* was the proportion value, and *n* was the sample size. We examined the coefficient comparing levels of treatment to determine significance in each case. The number of substrate licks was also examined using *glmmTMB*; however, as the duration of sessions was not fixed (range: 6 min 45 s to 60 min), we needed to ensure the response variable was considered as a rate (substrate licks per unit time). This was achieved by the inclusion of session duration (log transformed) as an offset in the model. As in the other models, we examined the coefficient comparing levels of treatment to determine significance. Submissive displays, tail raise and mouth open were rare and not analysed further (Figure A2).

The probability of a display occurring was analysed using the *glmer* function from the LME4 package (Bates et al. 2015), specifying a binomial error distribution. We set treatment as a fixed effect and lizard identity as a random effect and examined the coefficient comparing levels of treatment to determine significance. We then selected sessions in which a display occurred (*n* = 10 each for predator present/absent sessions) and compared aspects of the tail flicking component. The latency to tail flick, as well as the duration, time in motion and relative effort (Figure 1c) of the tail flicking component was examined using the *lme* function from the NLME package (Pinheiro et al. 2023). We used treatment as a fixed effect and lizard identity as a random effect, with response variables log transformed (except for latency to tail flick) to approximate a normal distribution. The statistical significance of each parameter was tested with *F* statistics. To facilitate comparisons with previous studies, we calculated effect sizes (*r*) and 95% confidence intervals for each variable using formula appropriate for mixed effects models as presented by Nakagawa and Cuthill (2007).

## 3 Results

**FIGURE 2:**
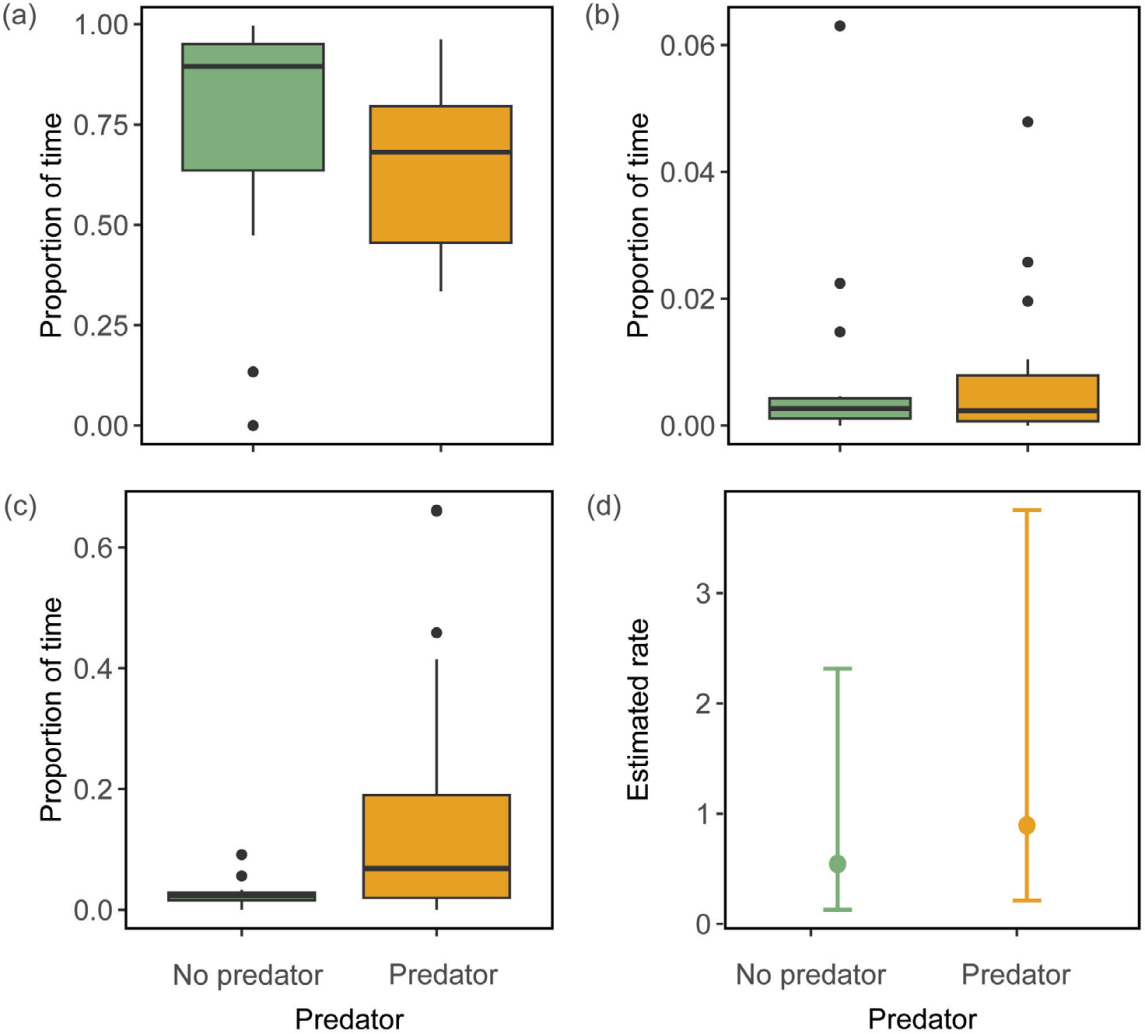
Mean ± SE proportion of time for (a) basking on an elevated perch, (b) moving about the enclosure and (c) eye directed upwards when the predator was present (orange bars) or absent (green bars). (d) Rate of substrate licking when the predator was present (orange point) or absent (green point).

**FIGURE 3:**
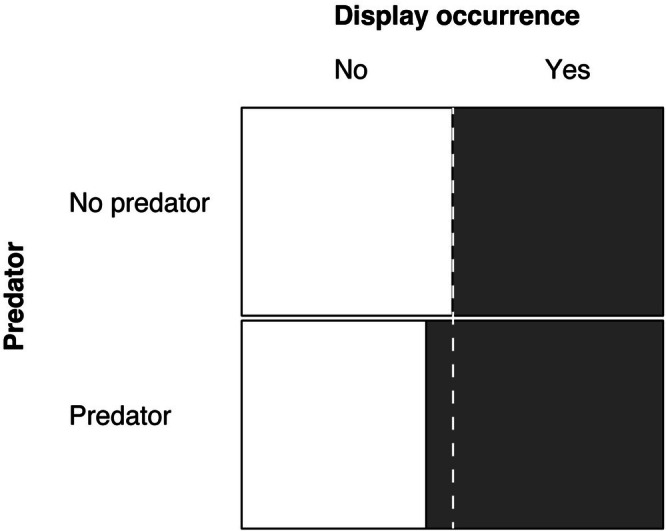
The proportion of sessions that did (grey) or did not (white) result in a display when the predator was present or absent.

**FIGURE 4:**
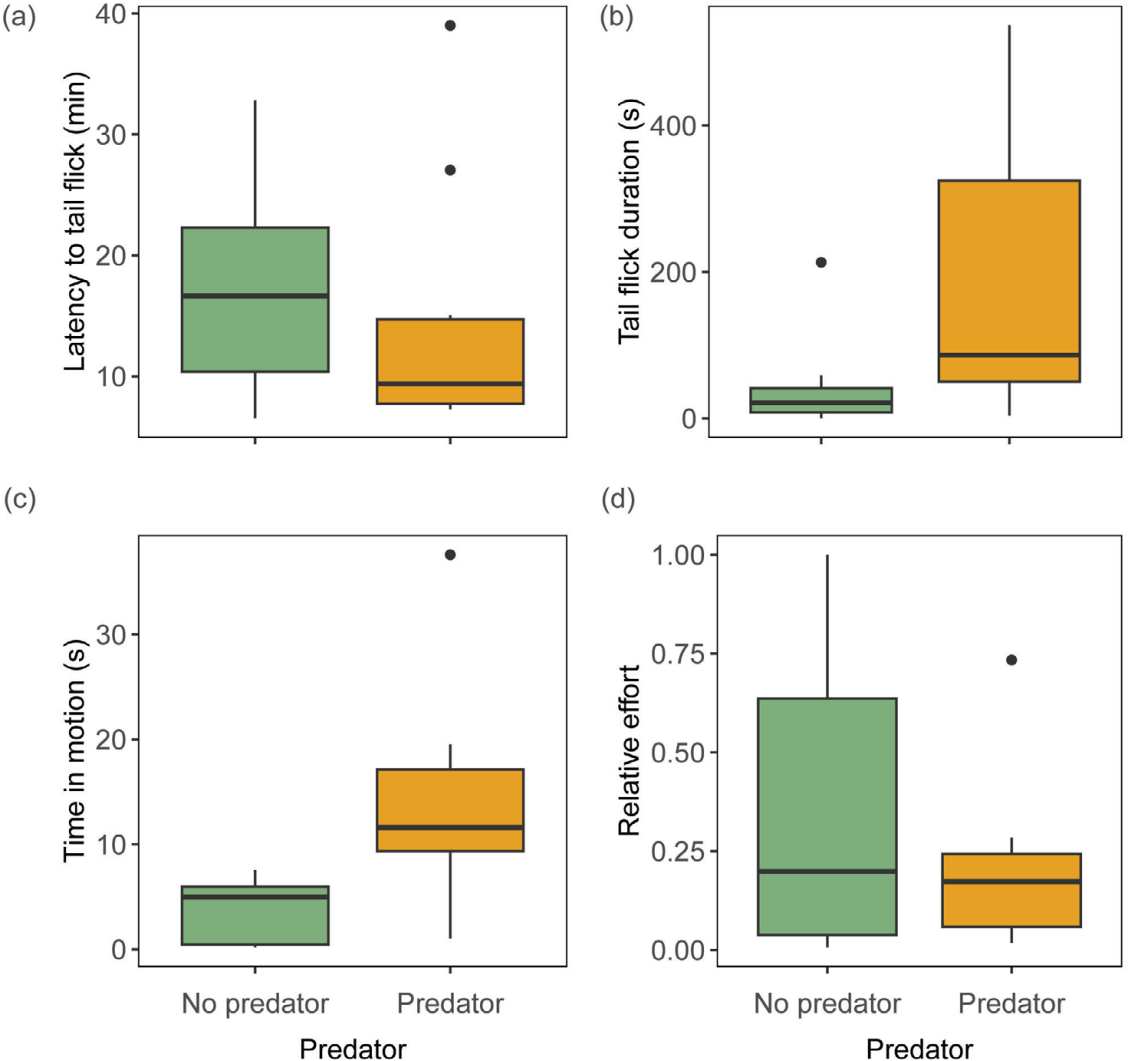
Mean ± SE for (a) latency to tail flick, as well as tail flick (b) duration, (c) time in motion and (d) relative effort when the predator was present (orange bars) or absent (green bars).

**FIGURE A3:**
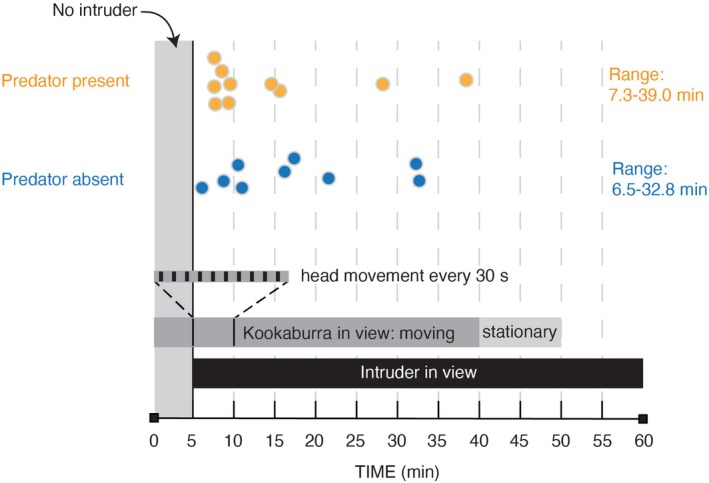
Lizards responded only after the intruder was released. Latency to first tail movement overlaid onto the illustration of the experimental design from Figure 1b. Individual response latencies are shown for the predator present (orange) and absent (blue) sessions. No responses were initiated prior to the release of the conspecific intruder.

Several aspects of lizard behaviour differed between predator‐present and predator‐absent conditions. The proportion of time spent basking on an elevated perch or moving in the enclosure did not vary between conditions (Figure 2a,b respectively; Table 1). However, lizards spent significantly more time with their eye up (Figure 2c; Table 1) and licked the substrate at a greater rate in the presence of a predator (Figure 2d; Table 1). The probability of generating an aggressive display was not influenced by the presence of a predator (Figure 3; Table 1), nor was there a difference in the latency to commence tail flicking (Figure 4a; Table 2), with all displays initiated after the intruder was revealed (Figure A3). Both the duration of tail flicking and time in motion were greater in the presence of the predator (Figure 4b,c), though only the latter was significant (Table 2). Extended time in motion is achieved through bouts of intermittent movement (Figure A3b), however, our measure of relative effort did not differ between treatments (Table 2); there was substantial variability in the predator absent condition while the predator present condition was less variable (Figure 4d).

**TABLE 1:** Results of regression models investigating difference in occurrence of different behaviours in sessions when the predator was not present compared to sessions when the predator was present.

Response variable in model	Estimate	SE	*z*‐score	*p*‐value
Basking	−0.360	0.372	−0.967	0.333
Moving	0.022	0.153	0.140	0.888
Eye up	**0.645**	**0.325**	**1.985**	**0.047**
Substrate licks	**0.498**	**0.230**	**2.163**	**0.031**
Probability of a display	0.570	1.087	0.524	0.600

*Note:* Significance (*p* < 0.05) is indicated in bold.

**TABLE 2:** Results of regression models investigating difference in the tail flicking component in sessions when the predator was not present compared to sessions when the predator was present.

Response variable in model	*F*‐statistic	DF	*p*‐value	Effect size	
				*r*	95% CI
Latency	0.842	1.7	0.389	−0.210	−0.531‐0.254
Duration	4.407	1.7	0.074	0.427	−0.046‐0.647
Time in motion	**8.817**	**1.7**	**0.021**	**0.539**	**0.099–0.702**
Relative effort	0.002	1.7	0.964	−0.011	−0.413‐0.398

*Note:* Significance (*p* < 0.05) is indicated in bold.

## 4 Discussion

Our findings provide further evidence that lizards are sensitive to the ecological context in which they signal. We found that predator presence alters the structure of introductory visual signals in *Amphibolurus muricatus* , resulting in an increase in the time in motion for the tail flick component rather than shortening signal duration. Lizards also spent significantly more time looking up toward the predator and licked the substrate at a greater rate in the presence of the predator, suggesting that they were aware of the predator's presence but showed no change in general activity or display likelihood. Taken together, these patterns indicate that individuals continued to engage in social signalling despite an elevated threat and, importantly, modified signal structure in a way that increased conspicuousness by increasing motion cues. This contradicts our initial prediction—derived from the assumption that predator‐induced plasticity primarily favours reduced detectability—and suggests that signalling decisions in *A. muricatus* reflect a more complex balance of costs and benefits.

**FIGURE 5:**
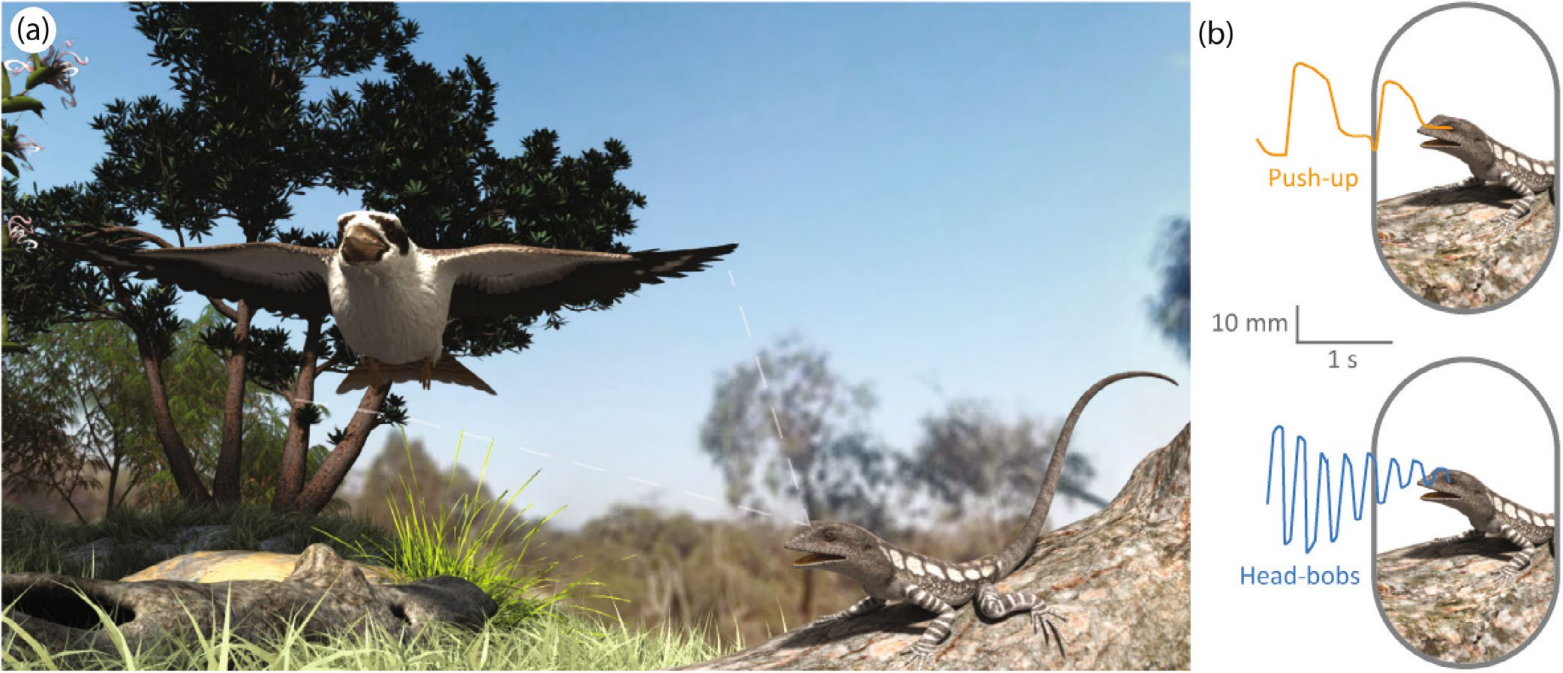
(a) Tail flicking ensures that the head and limbs are stationary while attempting to gain the attention of an intruder. The lizard's visual system is unaffected by self‐motion and can detect salient visual cues, such as an approaching predator. The limbs are also ready to initiate movement into cover of vegetation. (b) Alternative motor patterns centre on repeated movement of the head that might affect detection of approaching danger: Head movements (push‐ups) by *A. muricatus* and head‐bobbing by *Anolis sagrei* . Lines represent movement of the eye over time (display action pattern graphs). Unpublished data were used for *A. muricatus* while *A. sagrei* was redrawn from Steinberg et al. (2014).

Tail flicking employed by *A. muricatus* is well adapted to balancing communication benefits with survival demands. The intermittent nature of the tail flicking display reported here extends overall signal duration without continuous movement. This is crucial for two reasons: the effectiveness of tail flicking in eliciting an orienting response in receivers is mediated by signal duration (Peters and Evans 2003), and the stop‐start movement of the tail directly targets visual sensitivity to motion onsets and offsets, providing a reliable cue for the detection of motion (Ibbotson and Clifford 2001). Therefore, longer duration signals that directly target motion vision mechanisms will be more conspicuous than shorter duration continuous tail flicking. Another reason that tail flicking is ideal for signal elaboration is that it does not involve the head or limbs. The head remains stationary, stabilising potential threats within the visual field and preserving the ability to detect looming predators (Figure 5). At the same time, the limbs remain poised to initiate rapid escape to surrounding vegetation if danger is detected (Salisbury and Peters 2019). Thus, elaboration of tail flicking movements enhances attention and can be extended without compromising vigilance or escape readiness.

**FIGURE A4:**
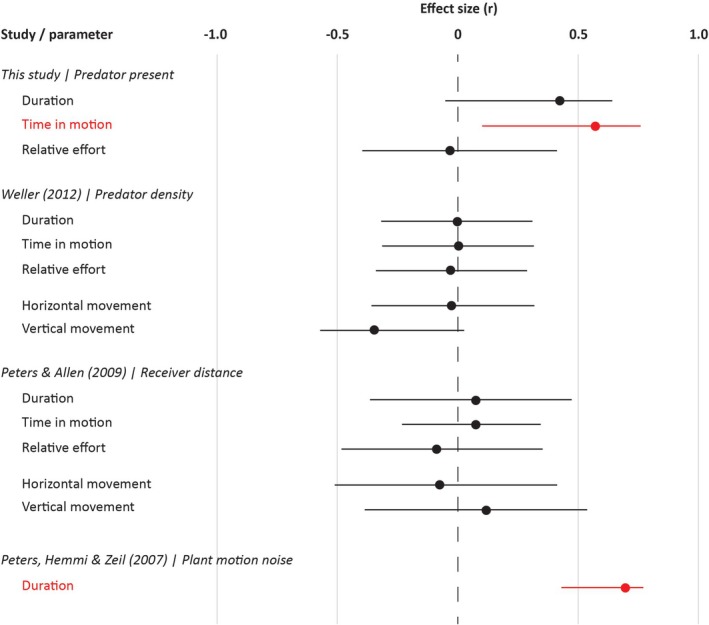
Effect sizes for all statistical analyses, along with previous studies for comparison. The temporal parameters from the present study are shown, along with preliminary study in which predator density was manipulated (Weller 2012). Also shown for comparison are effect sizes from two other studies that employed the same experimental design. The temporal and spatial parameters from Peters and Allen et al. (2009), which investigated the effect of receiver distance on signalling, and tail flick duration from Peters et al. (2007), which examined signal manipulation in response to environmental plant motion. Effect sizes and associated 95% confidence intervals were computed for mixed models as per Nakagawa and Cuthill (2007), and values in which the confidence interval does not overlap zero are highlighted in red.

Although increases in signal conspicuousness under predation risk have been reported in some contexts—such as compensating for visual motion noise, predator‐deterrent signals or deimatic displays—these explanations do not account for our findings. Previous studies have shown that lizards adjust signal structure to overcome background motion noise by increasing tail flick duration under sustained windy conditions (Peters et al. 2007). However, our results cannot be accounted for by signal‐to‐noise explanations as all plants were removed from enclosures, eliminating background motion. An alternative explanation for our findings is that lizards were signalling toward the predator rather than the conspecific. It is common for animals to signal toward predators to indicate they have been detected and to discourage an attack (Barbour and Clark 2012; Rao and Díaz‐Fleischer 2012; Caro 1995; Smythe 1970). Some lizards produce honest, conspicuous displays such as push‐ups to advertise condition and endurance and deter predator pursuit (Leal 1999; Leal and Rodriguez‐Robles 1997). In anticipation of this behaviour, our experimental design provided time between the appearance of the predator and the release of the intruder (Figure 1b). However, no signals commenced in this 5 min interval (Figure A3; Leal and Rodriguez‐Robles 1997), therefore, we do not consider this to account for our findings. Some lizards have evolved the use of deimatic displays to startle predators and increase chances of escape (Perez‐Martinez et al. 2020; Whiting et al. 2022), however, tail flicking in *A. muricatus* does not match deimatic display theory: it is prolonged rather than rapid and does not generate a sudden novel stimulus. Instead, we believe the data are consistent with the hypothesis of predator‐induced changes to signal structure in a way that increases conspicuousness, with an effect size for time in motion in the current study comparable to that for longer duration tail flicking produced by this species under conditions of increased environmental noise (Peters et al. 2007; Figure A4).

In most systems, predation risk is associated with reductions in conspicuous signalling (Simon 2007; Steinberg et al. 2014), consistent with the expectation that prey minimise informational accessibility to predators (Bernal and Page 2023). However, our results parallel cases in which individuals continue signalling when social or territorial benefits outweigh the immediate costs of increased exposure (Abbey‐Lee, Kaiser, et al. 2016; Akçay et al. 2016; Kelly and Godin 2001). Thus, animals flexibly adjust their signalling effort in response to both the risk and the need to communicate with conspecifics. Our study provides evidence that this dynamic also applies to the introductory stage of visual displays, where motion is used to secure attention before subsequent escalation. For *A. muricatus* , which occupies visually complex habitats and male behaviour consistent with territoriality, even modest reductions in signal efficacy are likely to compromise territory defence and mate access (Zwart et al. 2016). Conspicuous displays may therefore need to be maintained or even enhanced under risk. Ineffective communication between conspecifics during aggressive encounters could result in the loss of territory and subsequent reduction in mating opportunities (Mockford and Marshall 2009), and thus a reduction in reproductive fitness. Moreover, failure to resolve disputes through signalling may escalate encounters to physical combat, which carries an increased risk of detection and capture by predators (Jakobsson et al. 1995; Logue et al. 2010; Ota 2018). Therefore, increasing signal conspicuousness in the face of predation may in turn maximise the signaller's fitness if it allows them to retain the attention of conspecifics.

Interestingly, lizards increased their rate of substrate licks in the presence of the predator. Lizards use tongue‐flicking to sample chemical secretions on surfaces, allowing them to obtain olfactory cues about conspecifics and contextual information such as territory ownership (Campos et al. 2017). Substrate licking can provide information about intruding individuals and also function in self‐recognition by territory holders (Alberts 1992). Elevated rates of substrate licking have additionally been associated with exploratory behaviour in novel environments and under stressful conditions (De Fazio et al. 1977; Greenberg 1985). In the context of our study, increased rates of substrate licks may simply reflect stress‐induced arousal under heightened predation risk. Alternatively, it may represent an increased reliance on chemical information when visual attention is constrained by predator monitoring. Lizards may have been attempting to acquire more information about intruders via additional sensory modalities when visual systems were focused on the predator, as resident lizards spent more time looking up when the predator was present. Further research is needed to determine whether substrate licking under predation risk primarily reflects stress‐induced arousal or attempts to increase information acquisition via the use of alternative sensory cues.

We propose that the prolonged tail flicking component of the resident lizards' signalling display reflects a compensation for reduced receiver attention toward the signalling individual. Attentional mechanisms serve to focus on only a portion of sensory information available (Dukas 2002). Detecting and responding to stimuli is compromised if attention is diverted to other tasks (Clark and Dukas 2003), including within‐species social interactions (Yee et al. 2013). Predator presence likely redirects the receiver's vigilance toward threat monitoring, decreasing investment in social attention and compromising the efficacy of low‐intensity signals. Under this scenario, signallers increase motion and duration to maintain detectability and enhance the probability of an orienting response in intended receivers. As the behaviour of intruders was not monitored in this study, further research to test the reduced receiver attention hypothesis is needed to determine whether distracted receivers are delayed in providing cues to signallers that their presence is noted, or whether signallers anticipate that receivers are distracted and modify accordingly.

In summary, predator presence did not suppress signalling in *A. muricatus* ; instead, individuals increased the conspicuousness of introductory displays, consistent with the hypothesis that maintaining receiver attention can outweigh the risk imposed by a predator. More broadly, our results highlight that predator‐induced signal plasticity is multidirectional and may include increases in time in motion, and thus likely the conspicuousness when necessary to preserve communication function. These findings broaden current perspectives on predator‐induced signal plasticity and demonstrate that increases in conspicuousness, not just reductions, are a viable adaptive strategy under threat.

## Author Contributions

**Sonia Khanna:** conceptualization (equal), data curation (equal), formal analysis (lead), investigation (equal), methodology (supporting), project administration (supporting), validation (equal), visualization (equal), writing – original draft (lead), writing – review and editing (equal). **Richard A. Peters:** conceptualization (equal), data curation (equal), formal analysis (supporting), funding acquisition (lead), investigation (equal), methodology (lead), project administration (lead), supervision (lead), validation (equal), visualization (equal), writing – original draft (supporting), writing – review and editing (equal).

## Funding

This work was supported by the Australian Research Council, DP170102370.

## Ethics Statement

All procedures were approved by the Animal Ethics Committee of La Trobe University, Melbourne (AEC1043).

## Conflicts of Interest

The authors declare no conflicts of interest.

## Data Availability

Data is available at La Trobe University's Research Repository (OPAL) at the following DOI: https://doi.org/10.26181/32414334 (A temporary private is available during the review process: https://figshare.com/s/7175b3bf609f931814e1).

## Appendix 2

**TABLE A1:** Partial ethogram of captive *Amphibolurus muricatus* lizards.

Behaviour	Description
Basking (elevated)	Stationary on an elevated perch above ground, regardless of sun exposure
Basking	Stationary on the ground, regardless of sun exposure
Mouth open	Stationary with a single, wide opening of the mouth
Eye up	Head and eyes oriented upward toward the predator model
Moving	Locomotion involving movement of the body and/or limbs
Tail raise	Tail held in an upward curled arc resembling a semi‐circle
Aggressive display	Movement‐based signal comprising tail flicking and rapid sequence of limb and body movements (Display)
Tail flicking	The tail is moved, often with a stop‐start action, while the rest of the body is stationary
Tail move	Rapid movement of the tail originating from the tip (tail changes position between successive frames)
Tail stationary	Periods between tail movements during which the tail is motionless (no change in position of tail between successive frames)
Display	Sequence of motor patterns succeeding tail flicking, consisting of foreleg waves, followed by a push‐up, and a ‘body rock’ (the sequence often repeats)
Submissive display	Slow foreleg movement (either limb) in a circular motion
Substrate Lick	Movement of the tongue in and out of the mouth toward a substrate
